# Reliability of continuous cardiac output measurement during intra-abdominal hypertension relies on repeated calibrations: an experimental animal study

**DOI:** 10.1186/cc7102

**Published:** 2008-10-29

**Authors:** Matthias Gruenewald, Jochen Renner, Patrick Meybohm, Jan Höcker, Jens Scholz, Berthold Bein

**Affiliations:** 1 Department of Anaesthesiology and Intensive Care Medicine, University Hospital Schleswig-Holstein, Campus Kiel, Schwanenweg 21, D-24105 Kiel, Germany

## Abstract

**Introduction:**

Monitoring cardiac output (CO) may allow early detection of haemodynamic instability, aiming to reduce morbidity and mortality in critically ill patients. Continuous cardiac output (CCO) monitoring is recommended in septic or postoperative patients with high incidences of intra-abdominal hypertension (IAH). The aim of the present study was to compare the agreement between three CCO methods and a bolus thermodilution CO technique during acute IAH and volume loading.

**Methods:**

Ten pigs were anaesthetised and instrumented for haemodynamic measurements. Cardiac output was obtained using CCO by pulse power analysis (PulseCO; LiDCO monitor), using CCO by pulse contour analysis (PCCO; PiCCO monitor) and using CCO by pulmonary artery catheter thermodilution (CCO_PAC_), and was compared with bolus transcardiopulmonary thermodilution CO (CO_TCP_) at baseline, after fluid loading, at IAH and after an additional fluid loading at IAH. Whereas PulseCO was only calibrated at baseline, PCCO was calibrated at each experimental step.

**Results:**

PulseCO and PCCO underestimated CO, as the overall bias ± standard deviation was 1.0 ± 1.5 l/min and 1.0 ± 1.1 l/min compared with CO_TCP_. A clinically accepted agreement between all of the CCO methods and CO_TCP _was observed only at baseline. Whereas IAH did not influence the CO, increased CO following fluid loading at IAH was only reflected by CCO_PAC _and CO_TCP_, not by uncalibrated PulseCO and PCCO. After recalibration, PCCO was comparable with CO_TCP_.

**Conclusions:**

The CO obtained by uncalibrated PulseCO and PCCO failed to agree with CO_TCP _during IAH and fluid loading. In the critically ill patient, recalibration of continuous arterial waveform CO methods should be performed after fluid loading or before a major change in therapy is initiated.

## Introduction

Monitoring cardiac output (CO) allows early detection of haemodynamic instability and may be used to guide intensive care, aiming to reduce morbidity and mortality in high-risk patients [1]. In the past decade, continuous cardiac output (CCO) was commonly obtained by pulmonary artery catheter (PAC) with integrated heating filaments. The risk–benefit ratio of right heart catheterisation simply for CO determination has been questioned due to associated complications and the availability of less invasive alternatives [2]. Various monitor devices have been recently introduced into clinical practise that use the arterial pressure waveform to calculate CO on a beat-to-beat basis, such as the LiDCO™*plus *system using continuous cardiac output by pulse power analysis (PulseCO) and the PiCCO *plus *system using continuous cardiac output by pulse contour analysis (PCCO). Since arterial and central venous catheters are often already used to monitor critically ill patients, these techniques are not additionally invasive.

Several clinical studies have been performed on intensive care patients showing good agreement and correlation of the aforementioned methods of CCO determination with thermodilution or indicator-based techniques [3-10]. Some authors, however, recently questioned the reliability of these methods when acute changes of CO occur [11-14]. Therefore it is of high clinical interest to know whether the CCO method used is able to detect sudden CO changes, as frequently observed during haemorrhage, fluid loading or vasopressor administration [15]. Moreover, critically ill patients often present with intra-abdominal hypertension (IAH) [16]. Preliminary data by Malbrain and colleagues indicated unacceptable high limits of agreement of different invasive CCO measurements in 10 patients with IAH [17]. Reliability of CCO measurement during IAH and volume loading has not yet been elucidated in a controlled experimental setup. Increased intra-abdominal pressure is likely to modify several factors known to impact arterial waveform, such as chest wall compliance and arterial elastance, thereby potentially deteriorating agreement between the CO derived from thermodilution and derived from the arterial waveform.

The aim of the present study was to investigate whether PulseCO and PCCO – methods derived from the arterial waveform – and continuous assessment of continuous cardiac output by pulmonary artery catheter thermodilution (CCO_PAC_) are able to detect a volume-induced change of CO during IAH when compared with bolus transcardiopulmonary thermodilution cardiac output (CO_TCP_). Further, the level of agreement between these CCO values and CO_TCP _during IAH was determined. Finally, we analysed the impact of calibration on the accuracy of continuous beat-to-beat CO methods.

## Materials and methods

The present study was reviewed and approved by the local Animal Investigation Committee. The animals (10 healthy German domestic pigs, 58 ± 8 kg) were managed in accordance with our institutional guidelines, which are based on the *Guide for the Care and Use of Laboratory Animals *published by the National Institute of Health (NIH Publication No. 88.23, revised 1996).

The animals were fasted overnight, but had free access to water. The pigs were premedicated with the neuroleptic azaperone (4 to 8 mg/kg intramuscularly) and atropine (0.01 to 0.05 mg/kg intramuscularly) 1 hour before induction of anaesthesia with a bolus dose of ketamine (2 mg/kg intramuscularly), propofol (2 to 4 mg/kg intravenously) and sufentanil (0.3 μg/kg intravenously) given via an ear vein. After intubation with a cuffed endotracheal tube (internal diameter, 8.5 mm), the pigs were ventilated using a volume-controlled ventilator (Avea; Viasys Healthcare, Yorba Linda, CA, USA) with 10 ml/kg tidal volume, a positive end-expiratory pressure of 5 cmH_2_O, an inspiration:expiration ratio of 1:1.5 and a fraction of inspired oxygenof 0.35. The respiratory rate (12 to 18 breaths/min) was adjusted to maintain normocapnea (pressure of end-tidal CO_2 _= 35 to 45 mmHg). Oxygen saturation was monitored by a pulse oxymeter placed on the ear (M-CaiOV; Datex-Ohmeda, Helsinki, Finland).

Anaesthesia was maintained with a continuous infusion of propofol (6 to 8 mg/kg/hour) and sufentanil (0.3 μg/kg/hour), and muscle relaxation was provided by continuous infusion of pancuronium (0.2 mg/kg/hour) to ensure suppression of spontaneous gasping. In our experience, the animals do not respond to painful or auditory stimuli under this anaesthetic regimen when the paralysing agent is withheld, and the loading dose of ketamine and propofol subsides.

Ringer solution (5 ml/kg/hour) was administered during instrumentation. For induction of IAH, a Verres needle was inserted via a small infra-umbilical incision into the intra-abdominal cavity. The Verres needle was then connected to an electronic variable-flow insufflator (Wolf 2154701; Wolf GmbH, Knittlingen, Germany) for direct intra-abdominal pressure measurement and induction of IAH due to carbon dioxide pneumoperitoneum. The intra-abdominal pressure was measured in a supine position at end expiration.

### Cardiac output techniques

#### Pulse power analysis

PulseCO is a method integrated into the LiDCO™*plus *monitor (LiDCO™ Systems, London, UK). PulseCO uses pulse power analysis to determine the CCO by analysing the entire arterial waveform, and is not based on the morphology of the pulse contour. The system calculates the nominal stroke volume after a pressure-to-volume transformation using a curvilinear pressure/volume relationship. The nominal stroke volume is converted to the actual stroke volume by calibration of the algorithm based on lithium dilution using a bolus of 0.002 mmol/kg isotonic lithium chloride that was injected into the proximal port of the PAC. The lithium dilution curve was measured by a lithium ion-selective electrode (LiDCO, London, UK) located in a femoral arterial line, which was connected to the LiDCO device. Calibration of PulseCO was performed before muscle relaxation, because neuromuscular blockers may react with the lithium electrode.

#### Pulse contour analysis

PCCO is a method integrated into the PiCCO *plus *monitor (version 6.0; Pulsion Medical Systems, Munich, Germany). PCCO uses pulse contour analysis for calculation of the CCO and is based on a modified algorithm originally described by Wesseling and colleagues [18]. This algorithm enables continuous calculation of the stroke volume by measuring the systolic portion of the aortic pressure waveform and dividing the area under the curve by the individual aortic impedance. The PCCO device therefore needs to be calibrated by transcardiopulmonary thermodilution.

#### Continuous thermodilution by pulmonary artery catheter

CCO_PAC _is based on a semicontinuous pulsed warm thermodilution technique integrated into a PAC that is connected to a computer system (Vigilance Monitor; Baxter Edwards Critical Care, Irvine, CA, USA). The PAC (7.5-Fr Swan–Ganz CCO; Baxter Healthcare Corporation, Irvine, CA, USA) was inserted via an 8.5-Fr transducer into the right internal jugular vein for measuring the central venous pressure (CVP) and the pulmonary artery occlusion pressure (PAOP) and for CCO_PAC _recording.

#### Intermittent bolus transcardiopulmonary thermodilution

CO_TCP _is a bolus transcardiopulmonary thermodilution technique and served as the reference method and calibration method for PCCO. A 5-Fr thermistor-tipped arterial catheter (Pulsion Medical Systems) was inserted percutaneously into the right femoral artery, which was connected to the PiCCO *plus *monitor. A 10 ml bolus of cold (<8°C) saline was injected three times randomly assigned to the respiratory cycle into the proximal port of the PAC. Furthermore, an implemented algorithm enables calculation of the global end-diastolic volume (GEDV) as a volumetric variable of preload.

### Experimental protocol

**Figure 1:**
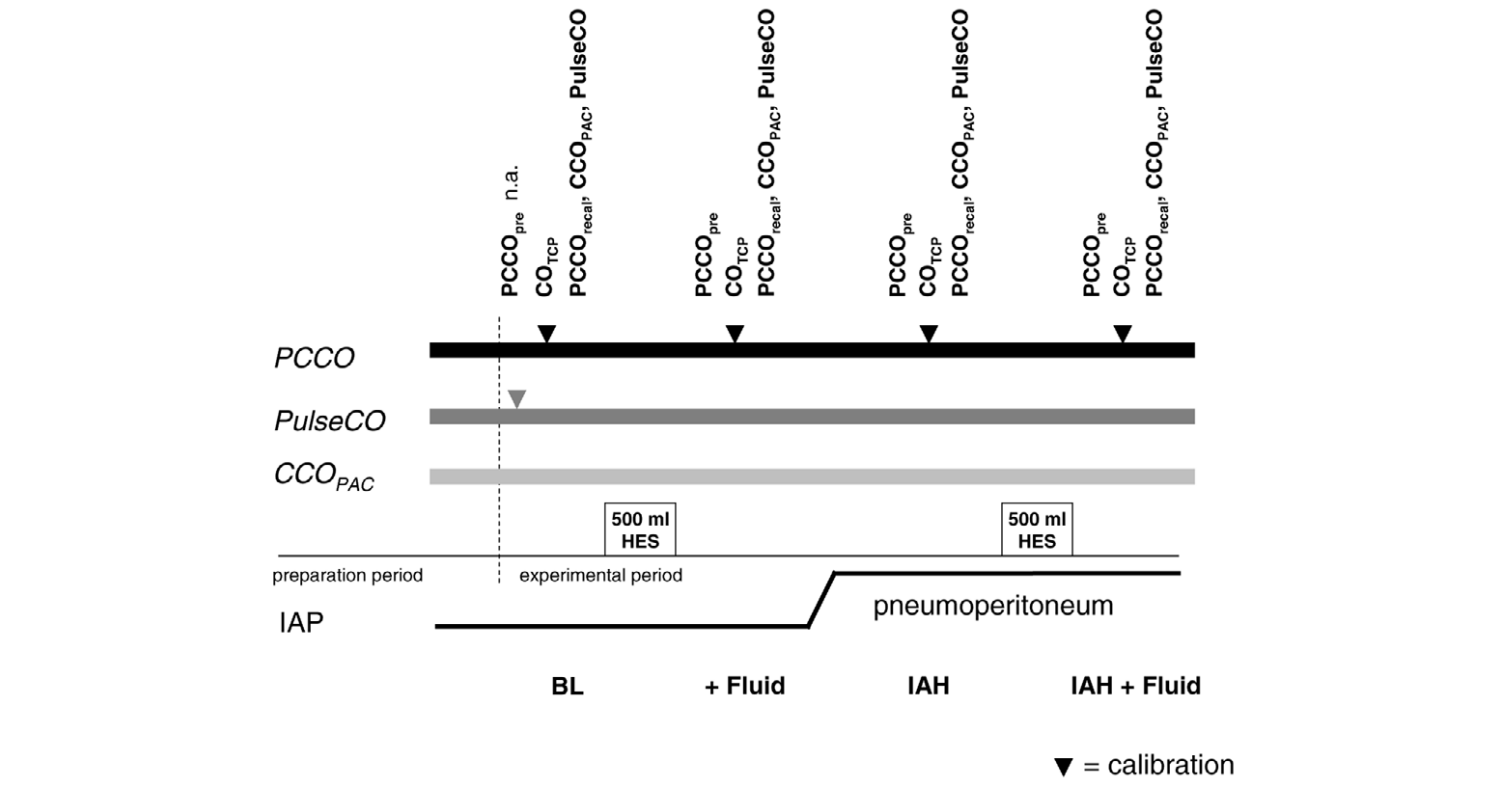
**Experimental protocol**. The methods used were continuous cardiac output by pulse contour analysis (PCCO; PiCCO system), continuous cardiac output by pulse power analysis (PulseCO; LiDCO system), continuous cardiac output by pulmonary artery catheter thermodilution (CCO_PAC_), and bolus transcardiopulmonary thermodilution cardiac output (CO_TCP_). PCCO was measured before recalibration (PCCO_pre_) and after recalibration (PCCO_recal_) by CO_TCP_. Experimental steps: BL, baseline; + Fluid, fluid loading; IAH, intra-abdominal hypertension; IAH + Fluid, second fluid load at IAH. HES, hydroxyl-ethyl starch 6%; IAP, intra-abdominal pressure; n.a., not applicable.

The experimental protocol is presented in Figure 1.

At the end of surgical preparation, at least 15 minutes were allowed for stabilisation. After taking baseline values, all animals received a fluid load of 500 ml hydroxyl-ethyl starch 6%. Equilibrium was expected after 10 minutes and measurements were repeated. Carbon dioxide was subsequently inflated into the abdominal cavity. IAH was assumed when the abdominal pressure was increased to at least 20 mmHg, reaching IAH grade III/IV according to the 2004 International Abdominal Compartment Syndrome Consensus Definitions Conference [19].

CO measurements were recorded after another stabilisation period of 10 minutes and again after a second fluid load of 500 ml hydroxyl-ethyl starch 6%. We recorded PCCO values 2 minutes before recalibration (PCCO_pre_) and 2 minutes after recalibration (PCCO_recal_) by CO_TCP _to control for a calibration effect. To avoid interference of CCO_PAC _with the bolus of ice-cold saline for CO_TCP _calibration, CO_TCP _was obtained at least 2 minutes in advance of CCO_PAC _recording. CCO_PAC _sampling was started after obtaining the CO_TCP_. PulseCO remained uncalibrated after baseline calibration throughout the experimental period. According to the manufacturer, calibration based on CO measured by lithium dilution or every other validated CO method is needed only once every 8 hours. PulseCO, PCCO and CCO_PAC _values were recorded and averaged during a period of 1 minute.

### Statistical analysis

Data are reported as the mean ± standard deviation (SD) unless otherwise specified. Statistical comparisons were performed using commercially available statistics software (GraphPad Prism 4; Graphpad Sofware Inc., San Diego, CA, USA).

Bland–Altman analysis was used to compare CO values by different measuring methods [20]. The bias represents the systemic error between two methods, and was defined as the mean difference between CO_A _and CO_B _values. Upper and lower limits of agreement, calculated as the bias ± 2 SDs, define the range in which 95% of the differences are expected to lie. The percentage error (PE) was calculated as reported by Critchley and Critchley [21], as limits of agreement (2 SD) divided by the mean CO from the two methods:

$$PE(\%)=\frac{2SD_{bias}}{(CO_{A}+CO_{B})/2}\cdot 100$$

In addition, data pairs were analysed using linear correlations and calculation of the coefficient of determination (*r*^2^). CO values after fluid loadings or initiation of IAH were compared using a paired *t *test. Furthermore, ΔCO was calculated as the percentage change of each CO method and was plotted against ΔCO_TCP _using linear regression and Bland–Altman analysis. *P *< 0.05 was considered statistically significant.

## Results

Nine animals were included in the final analysis. One pig was excluded from further analysis due to injury of the splenic vein by the Verres needle and a fatal outcome. All haemodynamic devices were installed and calibrated properly and no complications were associated with any of the devices. All animals were haemodynamically stable throughout the study period, no arrhythmias occurred, and no inotropic or antihypertensive drugs were administered. Pneumoperitoneum increased the intra-abdominal pressure by 17.7 ± 3.5 mmHg, and reduced chest wall compliance significantly by 64 ± 8%.

**Figure 2:**
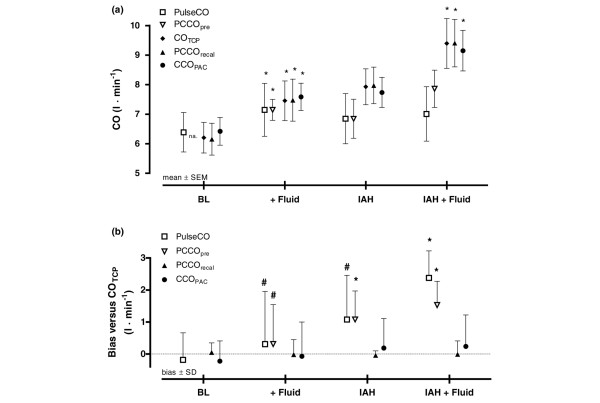
**Distribution and bias of cardiac output methods**. **(a) **Cardiac output (CO) measured by the different CO methods at each experimental step. **(b) **Bias and precision (standard deviation (SD)) between bolus transcardiopulmonary thermodilution cardiac output (CO_TCP_) and the different CO methods at each experimental step. PulseCO, continuous cardiac output by pulse power analysis (LiDCO system); PCCO, continuous cardiac output by pulse contour analysis (PiCCO system); CCO_PAC_, continuous cardiac output by pulmonary artery catheter thermodilution. PCCO was measured before recalibration (PCCO_pre_) and after recalibration (PCCO_recal_) by CO_TCP_. *P < 0.05 versus the previous experimental stage (PCCO_pre _versus previous PCCO_recal_). ^#^Methods not interchangeable according to Critchley and Critchley [21]. Filled symbols, calibrated measures. Experimental steps: BL, baseline; + Fluid, fluid loading; IAH, intra-abdominal hypertension; IAH + Fluid, second fluid load at IAH. IAP, intra-abdominal pressure; na, not applicable; SEM, standard error of the mean.

The haemodynamic variables are displayed in Table 1. The mean arterial pressure, GEDV, PulseCO, PCCO_pre_, PCCO_recal_, CO_TCP _and CCO_PAC _significantly increased after fluid loading at baseline, whereas the heart rate, CVP and PAOP remained unchanged. IAH significantly increased CVP and PAOP, but did not change the mean arterial pressure, heart rate, GEDV or CO values. Fluid loading during IAH did not significantly change the mean arterial pressure, heart rate, CVP, PAOP or GEDV, while CO_TCP_, CCO_PAC _and PCCO_recal _indicated a significant increase in CO. PulseCO and PCCO_pre_, however, were unable to reflect an increase of CO following fluid loading during IAH. Changes of CO determined by different methods throughout the experimental period are presented in Figure 2a. Individual time responses of each CO parameter are presented in detail (see Additional file 1, Figure S2).

**Table 1:** Haemodynamic data at each experimental step

Haemodynamic variable	Baseline	Fluid loading	IAH	IAH + Fluid
Mean arterial pressure (mmHg)	77 ± 20	99 ± 18*	109 ± 13*	112 ± 16*
Heart rate (beats/min)	87 ± 18	92 ± 18	92 ± 13	99 ± 12*
Central venous pressure (mmHg)	5 ± 2	7 ± 2	12 ± 4*^†^	12 ± 3*^†^
Pulmonary artery occlusion pressure (mmHg)	7 ± 2	11 ± 3	16 ± 6*^†^	16 ± 3*^†^
Intra-abdominal pressure (mmHg)	7 ± 2	7 ± 3	25 ± 3*^†^	27 ± 4*^†^
Chest wall compliance (ml/cmH_2_O)	58 ± 24	65 ± 22	22 ± 5*^†^	21 ± 4*^†^
Systemic vascular resistance (dyn·s/cm^5^)	963 ± 392	1006 ± 356	1038 ± 192	994 ± 303
Global end-diastolic volume (ml)	767 ± 164	883 ± 214*	928 ± 172*	997 ± 217*^†^
PulseCO (l/min)	6.4 ± 2.0	7.1 ± 2.7*	6.8 ± 2.5	7.0 ± 2.8
PCCO_pre _(l/min)	n.a.	7.1 ± 1.1*	6.8 ± 2.0	7.9 ± 1.9
CO_TCP _(l/min)	6.2 ± 1.6	7.5 ± 2.1*	8.0 ± 1.9*	9.4 ± 2.4*^†‡^
PCCO_recal _(l/min)	6.2 ± 1.6	7.5 ± 2.1*	8.0 ± 1.9*	9.4 ± 2.4*^†‡^
CCO_PAC _(l/min)	6.4 ± 1.4	7.6 ± 1.4*	7.7 ± 1.5*	9.1 ± 2.1*^†‡^

Data presented as the mean ± standard deviation. IAH, intra-abdominal hypertension; IAH + Fluid, second fluid load at IAH; PulseCO, continuous cardiac output by pulse power analysis; PCCO_pre_, continuous cardiac output by pulse contour analysis before calibration; CO_TCP_, bolus transcardiopulmonary thermodilution cardiac output; PCCO_recal_, continuous cardiac output by pulse contour analysis after recalibration; CCO_PAC_, continuous cardiac output by pulmonary artery catheter thermodilution. *P < 0.05 versus BL; ^†^*P *< 0.05 versus + Fluid; ^‡^*P *< 0.05 versus IAH (PCCO_pre _versus previous PCCO_recal_). n.a., not applicable.

Results of ΔCO comparison by Bland–Altman analysis and linear regression analysis are presented in Table 2 (for further details, see Additional file 1, Figure S1). ΔCCO_PAC _and ΔPCCO_recal _showed better agreement with ΔCO_TCP _than uncalibrated ΔPulseCO or ΔPCCO_pre_.

**Table 2:** Bland–Altman analysis and linear regression analysis of changes in cardiac output

	Bias (%)	Precision (%)	95% limits of agreement (%)	*r*^2^
ΔCO_TCP _and ΔPulseCO	12	19	-26 to 50	0.38
ΔCO_TCP _and ΔPCCO_pre_	14	18	-22 to 51	0.32
ΔCO_TCP _and ΔPCCO_recal_	-1	5	-10 to 9	0.94
ΔCO_TCP _and ΔCCO_PAC_	2	14	-26 to 30	0.47

Bias (mean difference), precision (standard deviation of bias), 95% limits of agreement, and correlation coefficient (*r*^2^) between changes (Δ) of CO measured by ΔPulseCO, ΔPCCO_pre_, ΔPCCO_recal _and ΔCCO_PAC _compared with ΔCO_TCP_. CO_TCP_, bolus transcardiopulmonary thermodilution cardiac output; PulseCO, continuous cardiac output by pulse power analysis; PCCO_pre_, continuous cardiac output by pulse contour analysis before calibration; PCCO_recal_, continuous cardiac output by pulse contour analysis after recalibration; CCO_PAC_, continuous cardiac output by pulmonary artery catheter thermodilution.

**Figure 3:**
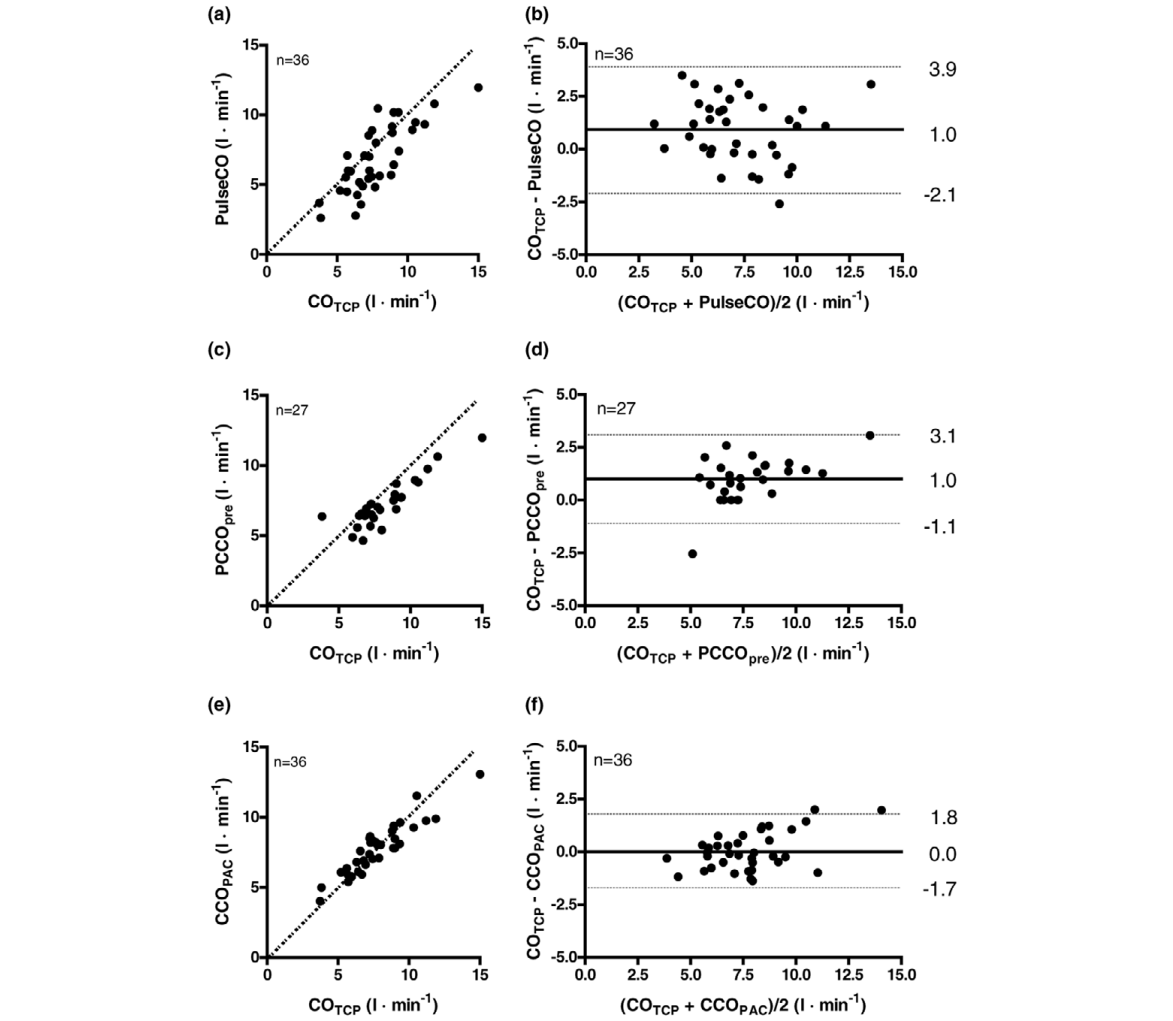
**Scatter plots and Bland–Altman plots of pooled data pairs**. Scatter plots (left-hand side) and Bland–Altman plots (right-hand side) of pooled data pairs between **(a) **and **(b) **bolus transcardiopulmonary thermodilution cardiac output (CO_TCP_) and continuous cardiac output by pulse power analysis (PulseCO; LiDCO system), **(c) **and **(d) **between CO_TCP _and continuous cardiac output by pulse contour analysis before recalibration (PCCO_pre_; PiCCO system), and **(e) **and **(f) **between CO_TCP _and continuous cardiac output by pulmonary artery catheter thermodilution (CCO_PAC_). (a), (c), (e) Scatter plots include line of identity (dotted line). (b), (d), (f) Bland–Altman plots include bias (solid lines) and limits of agreement (dotted lines).

Bland–Altman analysis revealed an overall (pooled data) bias ± SD (PE) between CO_TCP _and PulseCO of 1.0 ± 1.5 l/min (41.7%), between CO_TCP _and PCCO_pre _of 1.0 ± 1.1 l/min (27.5%), and between CO_TCP _and CCO_PAC _of 0.0 ± 0.9 l/min (23.3%). Figure 3a to 3f present Bland–Altman plots and correlation of pooled data comparing PulseCO, PCCO_pre _and CCO_PAC _with CO_TCP_.

The bias and precision (SD) between the examined CO methods and CO_TCP _at individual experimental steps are displayed in Figure 2b. The bias between CO_TCP _and the different CO methods was low at baseline, as criteria of interchangeability (PE <30%) were observed for all CO methods [21]. Fluid loading did not change the bias between methods significantly. After application of IAH, the bias between CO_TCP _and PulseCO and between CO_TCP _and PCCO_pre _increased, but this was only significant for PCCO_pre _(*P *< 0.05). Whereas fluid loading at baseline did not affect the bias between methods, the bias between CO_TCP _and PulseCO and between CO_TCP _and PCCO_pre _was significantly increased after fluid loading at IAH (*P *< 0.05). Calibration of PCCO_pre _reduced the bias significantly, as the bias between PCCO_recal _and CO_TCP _was low. Detailed results of Bland–Altman analysis and Pearson correlation comparisons between the different CCO methods and CO_TCP _at individual steps are available (see Additional file 1, Table S1).

## Discussion

The main findings of our experimental animal study are as follows. Firstly, at baseline without IAH, all CO methods showed acceptable agreement and reflected volume loading with an increase in CO. In contrast, IAH affects CO methods based on arterial waveform analysis in their ability to accurately indicate an increase in CO following fluid loading. Finally, recalibration of PCCO restored the system's accuracy.

The present study is the first on the agreement between three CCO methods and one intermittent bolus-thermodilution CO method during IAH and subsequent fluid loading. Research in the field of CCO monitoring has increased in recent years, as better evaluation of changes in a patient's haemodynamic status can facilitate therapy. Therefore it is of great interest whether these methods are able to reflect acute changes in CO induced by fluid loading under clinically relevant settings such as IAH.

Our results showed acceptable agreement of pooled CO data between CO_TCP_, PCCO_pre _+ PCCO_recal _and CCO_PAC_, whereas continuous beat-to-beat analysis by PulseCO calibrated only once underestimated the CO and failed interchangeability as defined by Critchley and Critchley [21].

With respect to CCO_PAC _versus CO_TCP _and CCO_PAC _versus PCCO, comparable agreement and correlation have been reported in several previous studies [22,23]. Compared with CO_TCP_, PCCO_recal _showed lower bias and lower PE than PCCO_pre_, a result expected intuitively. Volume loading in addition to IAH resulted in a significant increase of the CO measured by thermodilution techniques (CCO_PAC _and CO_TCP_). In contrast, beat-to-beat CO methods such as PulseCO and PCCO_pre _did not reflect the increase in CO accurately in the presence of IAH. The bias between PulseCO and PCCO_pre _versus CO_TCP _was significant after the second fluid load. Since the variability of bolus thermodilution is about 15%, we suggest that a mean bias within 15% of average CO can be clinically accepted. Recalibration of PCCO, as indicated by PCCO_recal_, results in a significant reduction of bias.

Taken together these findings emphasise that monitors tracking beat-to-beat CO benefit from frequent calibrations during changing loading conditions or changes of variables potentially influencing the underlying calculation algorithm, such as IAH.

All of the CO monitors showed clinically acceptable [21] agreement at baseline. The increase of CO due to fluid loading without IAH was also comparably reflected by all CO methods. The bias between methods remained unchanged. PulseCO failed interchangeability with CO_TCP_, however, as the PE increased clearly above 30% after fluid loading. Our data therefore suggest that PulseCO does not reliably indicate rapid haemodynamic changes following fluid loading. Similarly, Cooper and Muir recently reported PE >90% between PulseCO and lithium dilution CO after fluid resuscitation in haemorrhagic dogs [12].

While IAH reduced chest wall compliance and increased the CVP and PAOP, it did not significantly influence the CO. It is well known that cardiac filling pressures such as the CVP or PAOP in patients with IAH may be misleading, by falsely indicating increased preload [24]. Contrarily, preload during IAH may even be decreased due to substantial reductions in venous return, which is more pronounced in hypovolaemic patients. In the present study, however, the GEDV indicated normovolaemic conditions at baseline and no changes of preload due to IAH occurred. Consequently, it is not surprising that CO was not affected by IAH, which has been described previously [25].

There is still an ongoing debate about CO measurement derived from arterial waveform analysis and its ability to track changes of CO. In the present study, uncalibrated CCO methods were not able to reflect changes in CO appropriately. Several studies have shown good agreement of PulseCO with thermodilution or indicator-based CO methods in postsurgical intensive care patients [7-9]. On the other hand, Yamashita and colleagues reported that CO measured by PulseCO was not interchangeable with thermodilution during cardiac surgery [14]. Cooper and Muir [12] have shown only a moderate decline of PulseCO after induced haemorrhage in healthy dogs, with significant bias between PulseCO and lithium indicator dilution CO. The CO changes due to changes of intravascular volume might therefore not be adequately tracked by PulseCO. The authors concluded that false transformation of arterial pressure difference by PulseCO and changes of arterial compliance are possible explanations for the lack of accuracy to depict changes in CO. In the present study, PulseCO was only calibrated by the lithium dilution technique at baseline. Because of continuous application of neuromuscular blocking agents, a repeated calibration with lithium may be hampered due to interactions at the lithium electrode [9]. A calibration with another thermodilution technique is possible but this does not represent clinical practise and loses the advantage of being less invasive.

In contrast to PulseCO, PCCO was calibrated at each experimental step. A direct comparison of PCCO and PulseCO in the present study is therefore difficult. By calibrating PCCO repeatedly, it was already adjusted to the latest changes of vascular impedance. Interestingly, both PCCO_pre _and PulseCO underestimated CO after fluid loading in the presence of IAH with high bias compared with CO_TCP_. Our group recently reported high bias between uncalibrated PCCO and bolus pulmonary artery thermodilution CO in pigs during haemorrhage and norepinephrine administration [11]. In this study, uncalibrated PCCO did not reflect decreased CO as indicated by the PAC during a phlebotomy of almost 2 l – most probably due to failure to identify the dicrotic notch during a substantially increased heart rate. These findings were confirmed by Piehl and colleagues [26]. Additionally, Rodig and colleagues [2] reported a significant increase in bias between PCCO and CO_TCP _after cardiopulmonary bypass and vasopressor administration, most probably due to an increase in systemic vascular resistance. Sander and colleagues recommended frequent recalibration of PCCO after cardiopulmonary bypass due to changes in systemic vascular resistance [27]. In our study, however, systemic vascular resistance had no influence on the bias between methods.

The PCCO algorithm is based on the windkessel model by Otto Frank [28], including three major individual properties: aortic/arterial compliance, characteristic impedance, and peripheral vascular resistance. Calibration of PCCO by CO_TCP _enables the PCCO algorithm to correct for these three elements by calculating individual aortal compliance and systemic vascular resistance, and furthermore adjusting to aortic impedance. The ability of PCCO_pre _to accurately detect changes in CO due to fluid loading was hampered in the presence of IAH, however, whereas it was preserved at baseline. With respect to the effects of IAH, our results suggest that, due to reduced chest wall compliance, increased pleural and airway pressures are increasingly transmitted to the cardiac chambers, thereby reducing effective transmural pressure. Methods based on arterial waveform analysis are consequently prone to error in reflecting abrupt changes in CO, without an implemented algorithm to detect and correct for changes in vascular impedance as induced by IAH. The clinician therefore needs to consequently recalibrate the CCO based on arterial waveform analysis before any major change in therapy is initiated.

Our study has some limitations. The present study is an animal study and extrapolation to humans should be done with caution, and the reader should have this in mind. CCO_PAC _was obtained 2 minutes after bolus thermodilution, and hence a minor influence by recirculation of cold fluid is possible.

## Conclusion

All of the examined CO methods showed good agreement at baseline. There are limitations, however, in the ability of uncalibrated continuous CO methods based on arterial waveform analysis to accurately track changes in CO after fluid loading during IAH. The trend for underestimation of CO by PulseCO and PCCO_pre _documented in the present study could have clinical consequences. PCCO and PulseCO should be used with caution when assessing changes in CO after fluid loading, and should be recalibrated before any major change in therapy is initiated.

## Key messages

• CO measured by PulseCO, PCCO and CCO_PAC _showed good agreement with CO_TCP _without IAH, and reflected an increase in CO following fluid loading.

• Induction of IAH due to pneumoperitoneum did not significantly influence CO measured by PulseCO, PCCO, CCO_PAC _and CO_TCP_.

• At IAH, an increase in CO following fluid loading was indicated by calibrated PCCO, CCO_PAC _and CO_TCP _but not by uncalibrated CCO methods using arterial waveform analysis, such as PulseCO and PCCO.

• Recalibration of CCO parameters based on arterial waveform analysis should be done before any major change in therapy is initiated.

## Abbreviations

CCO: continuous cardiac output; CCO_PAC_: continuous cardiac output by pulmonary artery catheter thermodilution; CO: cardiac output; CO_TCP_: bolus transcardiopulmonary thermodilution cardiac output; CVP: central venous pressure; GEDV: global end-diastolic volume; IAH: intra-abdominal hypertension; PAC: pulmonary artery catheter; PAOP: pulmonary artery occlusion pressure; PCCO: continuous cardiac output by pulse contour analysis; PCCO_pre_: continuous cardiac output by pulse contour analysis before calibration; PCCO_recal_: continuous cardiac output by pulse contour analysis after recalibration; PE: percentage error; PulseCO: continuous cardiac output by pulse power analysis; SD: standard deviation.

## Competing interests

MG and JH declare that they have no competing interests. JR, PM, JS and BB have served as honorary lecturers for Pulsion Medical Systems, Inc.

## Authors' contributions

MG conceived of the study design, performed experiments, carried out statistical analysis and drafted the manuscript. JR conceived of the study design, carried out experiments and helped to draft the manuscript. PM and JH carried out data analysis and helped to draft the manuscript. JS coordinated the study. BB conceived of the study design, coordinated the study and helped with statistical analysis and drafting the manuscript. All authors read and approved the final manuscript.

## Supplementary Material

Additional file 1 Adobe file containing a table listing detailed results of Bland–Altman analysis comparing CO_TCP _and different CO methods at each individual step, Figure S1 showing linear regression and Bland–Altman plots comparing changes in CO (ΔCO) of different CO methods versus ΔCO_TCP_, and Figure S2 showing the individual time response of each CO parameter and each animal. Click here for file

## References

[B1] Eisenberg PR, Jaffe AS, Schuster DP (1984). Clinical evaluation compared to pulmonary artery catheterization in the hemodynamic assessment of critically ill patients. Crit Care Med.

[B2] Rodig G, Prasser C, Keyl C, Liebold A, Hobbhahn J (1999). Continuous cardiac output measurement: pulse contour analysis vs thermodilution technique in cardiac surgical patients. Br J Anaesth.

[B3] Bein B, Worthmann F, Tonner PH, Paris A, Steinfath M, Hedderich J, Scholz J (2004). Comparison of esophageal Doppler, pulse contour analysis, and real-time pulmonary artery thermodilution for the continuous measurement of cardiac output. J Cardiothorac Vasc Anesth.

[B4] Della Rocca G, Costa MG, Pompei L, Coccia C, Pietropaoli P (2002). Continuous and intermittent cardiac output measurement: pulmonary artery catheter versus aortic transpulmonary technique. Br J Anaesth.

[B5] Buhre W, Weyland A, Kazmaier S, Hanekop GG, Baryalei MM, Sydow M, Sonntag H (1999). Comparison of cardiac output assessed by pulse-contour analysis and thermodilution in patients undergoing minimally invasive direct coronary artery bypass grafting. J Cardiothorac Vasc Anesth.

[B6] Bottiger BW, Sinner B, Motsch J, Bach A, Bauer H, Martin E (1997). Continuous versus intermittent thermodilution cardiac output measurement during orthotopic liver transplantation. Anaesthesia.

[B7] Pittman J, Bar-Yosef S, SumPing J, Sherwood M, Mark J (2005). Continuous cardiac output monitoring with pulse contour analysis: a comparison with lithium indicator dilution cardiac output measurement. Crit Care Med.

[B8] Hamilton TT, Huber LM, Jessen ME (2002). PulseCO: a less-invasive method to monitor cardiac output from arterial pressure after cardiac surgery. Ann Thorac Surg.

[B9] Costa MG, Della Rocca G, Chiarandini P, Mattelig S, Pompei L, Barriga MS, Reynolds T, Cecconi M, Pietropaoli P (2008). Continuous and intermittent cardiac output measurement in hyperdynamic conditions: pulmonary artery catheter vs. lithium dilution technique. Intensive Care Med.

[B10] Linton NW, Linton RA (2001). Estimation of changes in cardiac output from the arterial blood pressure waveform in the upper limb. Br J Anaesth.

[B11] Bein B, Meybohm P, Cavus E, Renner J, Tonner PH, Steinfath M, Scholz J, Doerges V (2007). The reliability of pulse contour-derived cardiac output during hemorrhage and after vasopressor administration. Anesth Analg.

[B12] Cooper ES, Muir WW (2007). Continuous cardiac output monitoring via arterial pressure waveform analysis following severe hemorrhagic shock in dogs. Crit Care Med.

[B13] Hamzaoui O, Monnet X, Richard C, Osman D, Chemla D, Teboul JL (2008). Effects of changes in vascular tone on the agreement between pulse contour and transpulmonary thermodilution cardiac output measurements within an up to 6-hour calibration-free period. Crit Care Med.

[B14] Yamashita K, Nishiyama T, Yokoyama T, Abe H, Manabe M (2005). Cardiac output by PulseCO is not interchangeable with thermodilution in patients undergoing OPCAB. Can J Anaesth.

[B15] Michard F (2007). Pulse contour analysis: fairy tale or new reality?. Crit Care Med.

[B16] Malbrain ML, Chiumello D, Pelosi P, Wilmer A, Brienza N, Malcangi V, Bihari D, Innes R, Cohen J, Singer P, Japiassu A, Kurtop E, De Keulenaer BL, Daelemans R, Del Turco M, Cosimini P, Ranieri M, Jacquet L, Laterre PF, Gattinoni L (2004). Prevalence of intra-abdominal hypertension in critically ill patients: a multicentre epidemiological study. Intensive Care Med.

[B17] Malbrain M, De Iaet I, Viaene D, Vermeiren G, Schoonheydt K, Dits H (2007). Validation of four different continuous minimal invasive cardiac output methods in patients with abdominal hypertension. Acta Clin Belg Suppl.

[B18] Wesseling KH, Jansen JR, Settels JJ, Schreuder JJ (1993). Computation of aortic flow from pressure in humans using a nonlinear, three-element model. J Appl Physiol.

[B19] Cheatham ML, Malbrain ML, Kirkpatrick A, Sugrue M, Parr M, De Waele J, Balogh Z, Leppaniemi A, Olvera C, Ivatury R, D'Amours S, Wendon J, Hillman K, Wilmer A (2007). Results from the International Conference of Experts on Intra-abdominal Hypertension and Abdominal Compartment Syndrome. II. Recommendations. Intensive Care Med.

[B20] Bland JM, Altman DG (1986). Statistical methods for assessing agreement between two methods of clinical measurement. Lancet.

[B21] Critchley LA, Critchley JA (1999). A meta-analysis of studies using bias and precision statistics to compare cardiac output measurement techniques. J Clin Monit Comput.

[B22] Spohr F, Hettrich P, Bauer H, Haas U, Martin E, Bottiger BW (2007). Comparison of two methods for enhanced continuous circulatory monitoring in patients with septic shock. Intensive Care Med.

[B23] Della Rocca G, Costa MG, Coccia C, Pompei L, Di Marco P, Vilardi V, Pietropaoli P (2003). Cardiac output monitoring: aortic transpulmonary thermodilution and pulse contour analysis agree with standard thermodilution methods in patients undergoing lung transplantation. Can J Anaesth.

[B24] Cheatham ML, Safcsak K, Block EF, Nelson LD (1999). Preload assessment in patients with an open abdomen. J Trauma.

[B25] Sumpelmann R, Schuerholz T, Marx G, Jesch NK, Osthaus WA, Ure BM (2006). Hemodynamic changes during acute elevation of intra-abdominal pressure in rabbits. Paediatr Anaesth.

[B26] Piehl MD, Manning JE, McCurdy SL, Rhue TS, Kocis KC, Cairns CB, Cairns BA (2008). Pulse contour cardiac output analysis in a piglet model of severe hemorrhagic shock. Crit Care Med.

[B27] Sander M, von Heymann C, Foer A, von Dossow V, Grosse J, Dushe S, Konertz WF, Spies CD (2005). Pulse contour analysis after normothermic cardiopulmonary bypass in cardiac surgery patients. Crit Care.

[B28] Sagawa K, Lie RK, Schaefer J (1990). Translation of Otto Frank's paper ¸Die Grundform des Arteriellen Pulses' Zeitschrift fur Biologie 37: 483–526 (1899). J Mol Cell Cardiol.

