# Supplementary material for: Reliability of continuous cardiac output measurement during intra-abdominal hypertension relies on repeated calibrations: an experimental animal study
Source: Crit Care. 2008 Oct 29;12(5):R132. doi: 10.1186/cc7102 (PMC2592771; doi:10.1186/cc7102)
Supplement: Additional file 1 — Adobe file containing a table listing detailed results of Bland–Altman analysis comparing COTCP and different CO methods at each individual step, Figure S1 showing linear regression and Bland–Altman plots comparing changes in CO (ΔCO) of different CO methods versus ΔCOTCP, and Figure S2 showing the individual time response of each CO parameter and each animal. [file cc7102-S1.pdf]

Additional data file

Table S1: Bland-Altman Analysis comparing CO_{TCP} and different CO methods

CO_{TCP}-	$CO_{average}$ ($l\cdot min^{-1}$)	bias ± SD ($l\cdot min^{-1}$)	95% LoA ($l\cdot min^{-1}$)	PE (%)	r^2
**all (pooled data)**					
PulseCO	7.3 ± 2.1	1.0 ± 1.5	-2.1 – 3.9	41.7 #	0.63
$PCCO_{pre}$	7.8 ± 1.9	1.0 ± 1.1	-1.1 – 3.1	27.5	0.78
$PCCO_{recal}$	7.8 ± 2.3	0.0 ± 0.3	-0.7 – 0.6	8.7	0.98
CCO_{PAC}	7.7 ± 2.0	0.0 ± 0.9	-1.7 – 1.8	23.3	0.85
**Baseline**					
PulseCO	6.3 ± 1.7	-0.2 ± 0.8	-1.8 – 1.5	25.4	0.85
$PCCO_{pre}$	n.a.	n.a.	n.a.	n.a.	n.a.
$PCCO_{recal}$	6.2 ± 1.6	0.0 ± 0.3	-0.5 – 0.6	9.7	0.90
CCO_{PAC}	6.3 ± 1.4	-0.2 ± 0.6	-1.5 – 1.0	19.9	0.83
**+ Fluid**					
PulseCO	7.3 ± 2.2	0.3 ± 1.6	-2.8 – 3.4	43.8 #	0.65
$PCCO_{pre}$	7.3 ± 1.5	0.3 ± 1.2	-2.1 – 2.7	33.7 #	0.73
$PCCO_{recal}$	7.5 ± 2.0	0.0 ± 0.5	-0.9 – 0.9	12.5	0.95
CCO_{PAC}	7.5 ± 1.6	-0.1 ± 1.1	-2.2 – 2.0	28.6	0.71
**IAH**					
PulseCO	7.4 ± 2.1	1.1 ± 1.4	-1.6 – 3.8	38.8 #	0.73
$PCCO_{pre}$	7.4 ± 1.8	1.1 ± 0.9 *	-0.7 – 2.8	24.1	0.80
$PCCO_{recal}$	8.0 ± 1.8	0.0 ± 0.1	-0.3 – 0.2	3.5	0.99
CCO_{PAC}	7.8 ± 1.6	0.2 ± 0.9	-1.6 – 2.0	23.5	0.75
**IAH + Fluid**					
PulseCO	8.2 ± 2.6	2.4 ± 0.8 *	0.7 – 4.0	20.5	0.91
$PCCO_{pre}$	8.6 ± 2.2	1.5 ± 0.7 *	0.1 – 3.0	17.2	0.97
$PCCO_{recal}$	9.4 ± 2.4	0.0 ± 0.4	-0.8 – 0.8	8.9	0.97
CCO_{PAC}	9.3 ± 2.3	0.2 ± 1.0	-1.7 – 2.2	21.1	0.86

* $p<0.05$ vs. bias at previous experimental stage ($PCCO_{pre}$ vs. previous $PCCO_{recal}$)

# not interchangeable according to Critchley et al.

presented are mean difference (bias) ± standard deviation (SD) together with limits of agreement (LoA), mean percentage error (PE) and coefficient of correlation (r^2) comparing different methods and CO_{TCP}, of all (pooled) data sets and data at different experimental steps.

PulseCO (pulse power analysis cardiac output, LiDCO system), PCCO (pulse contour cardiac output, PiCCO system), CCO_{PAC} (continuous pulmonary artery catheter cardiac output), CO_{TCP} (bolus trans-cardiopulmonary thermodilution cardiac output). PCCO was measured before ($PCCO_{pre}$) and after ($PCCO_{recal}$) recalibration by CO_{TCP}

Figure S1: linear regression and Bland-Altman plots comparing changes (Δ) in CO

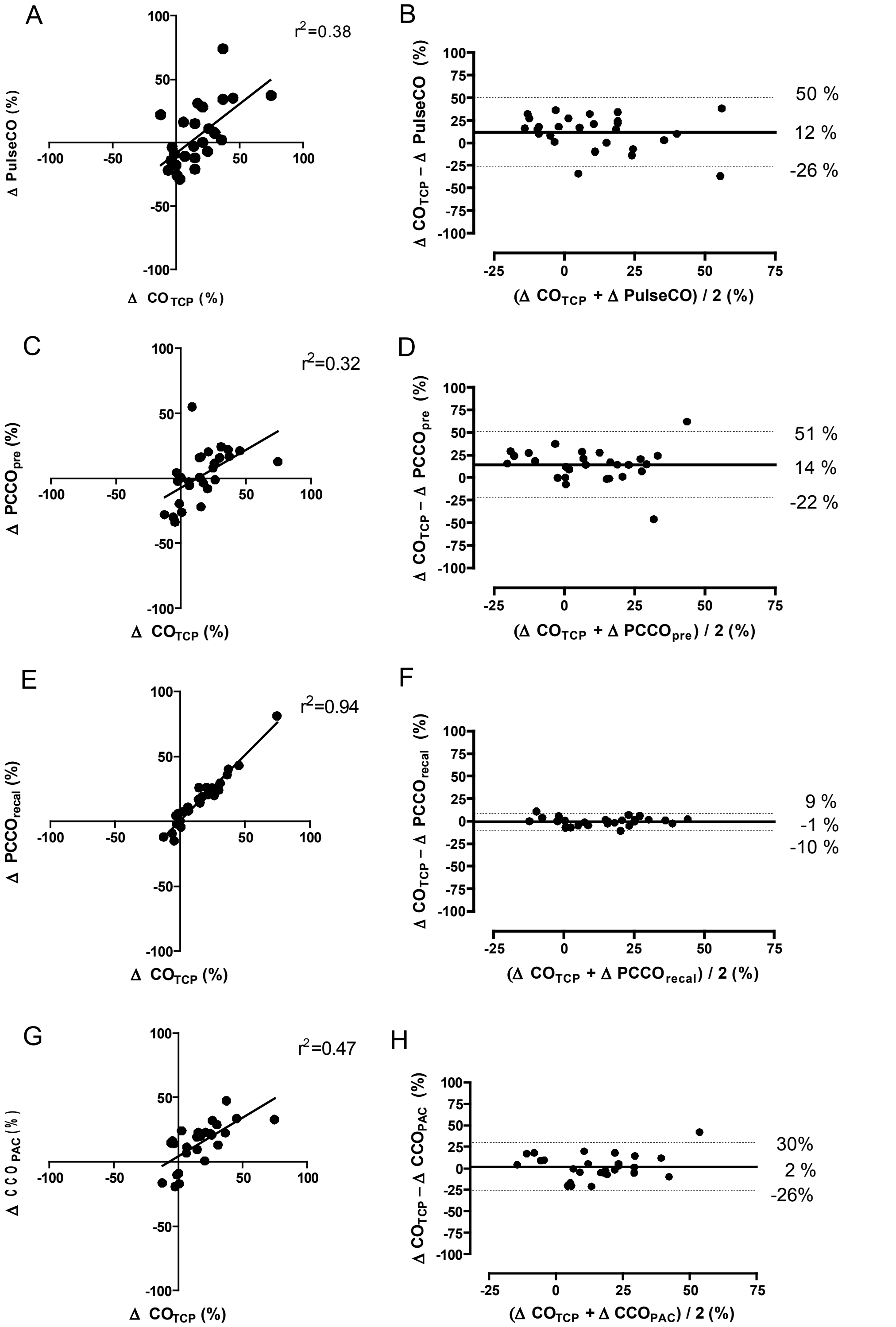

Figure S1: Linear regression (left) and Bland-Altman plots (right) comparing

A, B) PulseCO vs. CO_{TCP}; C, D) $PCCO_{pre}$ vs. CO_{TCP}; E, F) $PCCO_{recal}$ vs. CO_{TCP};

G, H) CCO_{PAC} vs. CO_{TCP}

left: regression line – solid line right: bias - solid line (± 2SD – dotted lines)

Figure S2: Individual time response of each CO parameter and each animal

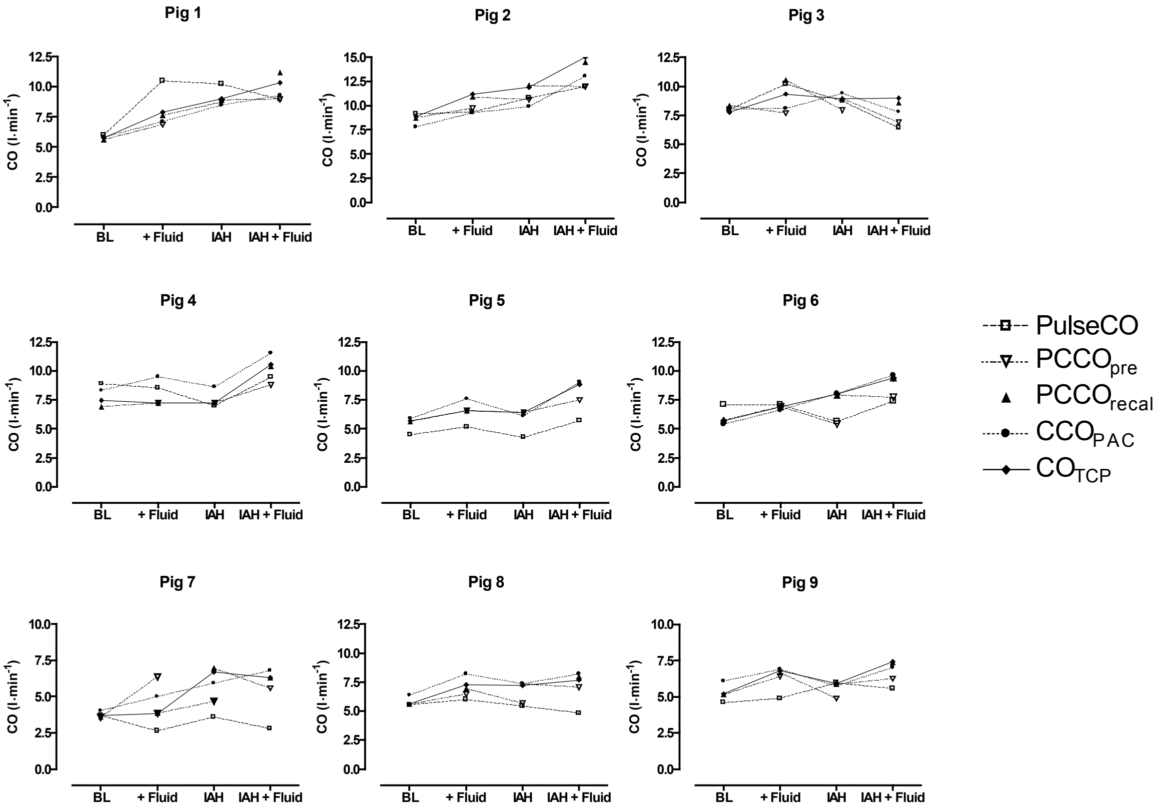

Figure S2: Individual time response of each CO parameter and each animal

Experimental steps: BL - baseline; + Fluid - fluid loading; IAH - intra-abdominal hypertension, IAH + Fluid - second fluid load at IAH

PulseCO (pulse power analysis cardiac output, LiDCO system), PCCO (pulse contour cardiac output, PiCCO system), CCO_{PAC} (continuous pulmonary artery catheter cardiac output), CO_{TCP} (bolus trans-cardiopulmonary thermodilution cardiac output). PCCO was measured before ($PCCO_{pre}$) and after ($PCCO_{recal}$) recalibration by CO_{TCP}.
